# Scenes in the Busiest Week of the War

**Published:** 1914-10-24

**Authors:** 


### BRITISH AND BELGIAN WOUNDED AT CARDIFF.

### Scenes in the Busiest Week of the War.

Cardiff last week witnessed scenes of a kind unknown in its history by the arrival of both British and Belgian wounded soldiers and their meeting in the various military hospitals. Last week was the busiest since the war, for on Tuesday 110 of the men from the Front were conveyed direct from the steamer at Southampton to a train which left that port at 10.30 A.M. and reached Cardiff after a four-hours' journey. The train consisted of nine brake-vans, specially adapted for ambulance purposes by the Great Central Railway Company.

On the strictly reserved platform were Colonel East, D.S.O., in command of the Severn Defences, Major Ewen Maclean, M.D., Adjutant and Registrar of the 3rd Western General Hospital, Major-General Lee, chairman of the board of management of King Edward VII.'s Hospital, and Major C. W. Jordan in charge of the Motor Transport Service.

The Glamorgan Red Cross Society sent five squads from the 1st Voluntary Aid Detachment (Cardiff), four squads from Barry, four from St. Fagan's and Dinas Powis, and three from Penarth to assist the wounded from the train to the motor vans, cars, and chars-à-bancs, which were in charge of Dr. Sparrow of Cardiff. An interesting feature was the keeping of the approaches by about fifty of the Cardiff Special Police, consisting of professional and business men, who had attended at but two hours' notice. They were in charge of the Head Constable, Mr. David Williams.

At 2.30 the train, with the familiar red cross, steamed into the station. Lieutenant Wolstenholme, R.A.M.C., was in charge of the wounded, and on alighting at once gave the list of names to Major Maclean. The work of detraining was accomplished in half an hour.

As the first soldier—a Highlander, supporting himself on a crutch, with one foot heavily bandaged—emerged, a great cheer arose. Then followed the others, generally in batches of twos and threes, most of them limping or with head or arm bandaged. But some were obviously seriously hurt, and required tender handling by the ambulance men. One noted their soiled, stained, and torn clothing and the condition of their boots, telling in eloquent tatters of the ordeal through which they had lately passed.

The men did not carry the customary trophies of war, but the grimness of the scene was relieved somewhat by the adornments of heather, violets, and other flowers, which had been given to them at Trowbridge and other places *en route*. Those who were able waved their hands or lifted their caps in happy acknowledgment of their splendid reception. The arrangements made by Colonel Hepburn, V.D., and his staff for receiving, secured that within an hour of their arrival at Cardiff they were all comfortably settled—85 were "sitting-up" cases and 25 "lying-down" or "cot." The medical cases were 44 and the surgical 66, some of the latter having already been operated upon before being sent from Southampton. Among the former was a high percentage of men suffering from rheumatism. They were allotted to the hospitals as follows:—Splott Road Council Schools, 54; Albany Road Council Schools, 33; King Edward VII.'s Hospital, 23.

On Friday last week there reached Cardiff 235 wounded Belgian soldiers who had come from the fighting line at Antwerp. They travelled *via* Ostend to Southampton, and were brought to the Glamorgan capital in an ambulance train which arrived at 9.30 A.M. Numbers of them had arms or heads bandaged, but the majority had wounds in legs and feet, the latter being carried by the Red Cross men. There were only twenty "cot" cases, and these were conveyed on stretchers to the motor ambulances.

With uniforms rough, nondescript, stained, and dirt-covered, faces unshaven, they passed along in motor-cars amid enthusiastic scenes. There was a rush for caps, buttons, and various other mementoes, and one young soldier with a badly damaged leg exchanged his military cap for the cap of a newspaper employee, the object of the latter being to raffle it at the office for the benefit of the refugees! The ever-welcome cigarette was scattered broadcast in return. The men were taken to three of the sections of the 3rd Western General Hospital: 140 to Howard Gardens Schools; 40 to Splott Road Schools; and 55 to Albany Road Schools. They are loud in their praise of the treatment which they are receiving on every hand.

### Two Stirring Stories.

They relate stirring stories, of which the two following are examples:

Private Yanssen, 6th Line Regiment, 3rd Division, 3rd Company, had been in six different engagements. At Wavre St. Catherine he got his first wound when sent out with six men to prevent the Germans from cutting the barbed wires. They killed about thirty Germans that night, but he got badly burned with the deadly shrapnel, and was in hospital twelve days. On the thirteenth day he was again in the battlefield. He later fought at Aerschot and in the villages around it. At Louvain he sat thirty-seven hours in a trench where it rained all night, and the water was 2 feet deep in the trench in the morning. He was stiff with rheumatism.

The other story was told by Private Charles Delbeke, of the 1st Regiment of the Line. About a fortnight ago his regiment was sent to Buggenhout as reinforcements. They were encamped in a wood, and about midnight shells started screaming over them. Till 5 A.M. they had to lie quiet, and then they crawled along in ditches in small detachments to discover where the Germans were concealed. A corporal and three men went ahead whilst the rest entrenched themselves, and five minutes later only two returned, stating that the Germans' position was on the left. Again the enemy commenced shelling the Belgian trenches, forcing them to retreat about 500 yards. Then their captain—a man who suffered from acute bronchial trouble—accompanied by twelve men, made a dash for a part of the German position in the attempt to silence one of their guns. But as soon as they came out of the trench a hail of lead was poured on them, killing eight men. The rest ran for their lives, and the gallant captain was so exhausted that he had to lay down flat in the trench and was covered with straw. A sergeant then took command, and said, "Now, boys, we've got to succeed or die; we must go for them for all we are worth." They thereupon attempted to reach the Germans by running along in a deep ditch with water up to their waists. In the race they had to bend down so as to avoid the bullets flying overhead. It was certain death to show one's head and shoulders, but several did so, and down they splashed dead into the water, and their comrades raced on, jumping over the corpses. Private Delbeke was struck in the back of the neck with a bullet, but it did not injure a vital part, and passed out under the jaw. He fell on his knees, struggled to his feet again, when another bullet lodged in his shoulder, and he fainted away. He did not know the result of the charge, as he did not regain consciousness until he reached hospital.

